# Experience conducting COVID-19 vaccine effectiveness studies in response to the COVID-19 pandemic in Japan and the Philippines: lessons for future epidemics and potential pandemics

**DOI:** 10.5365/wpsar.2025.16.2.1157

**Published:** 2025-06-04

**Authors:** Takeshi Arashiro, Regina Pascua Berba, Joy Potenciano Calayo, Rontgene Solante, Shuichi Suzuki, Jinho Shin, Motoi Suzuki, Martin Hibberd, Koya Ariyoshi, Chris Smith

**Affiliations:** a Faculty of Infectious and Tropical Diseases, London School of Hygiene and Tropical Medicine, London, United Kingdom of Great Britain and Northern Ireland.; b School of Tropical Medicine and Global Health, Nagasaki University, Nagasaki, Japan.; c Center for Surveillance, Immunization, and Epidemiologic Research, National Institute of Infectious Diseases, Tokyo, Japan.; d Department of Pathology, National Institute of Infectious Diseases, Tokyo, Japan.; e World Health Organization Regional Office for the Western Pacific, Manila, Philippines.; f Hospital Infection Control Unit, Philippine General Hospital, Manila, Philippines.; g Department of Laboratory, San Lazaro Hospital, Manila, Philippines.; h Adult Infectious Diseases and Tropical Medicine Unit, San Lazaro Hospital, Manila, Philippines.; i San Lazaro Hospital-Nagasaki University Collaborative Research Office and Laboratory, San Lazaro Hospital, Manila, Philippines.

## Abstract

**Problem:**

Once COVID-19 vaccines were rolled out, there was a need to monitor real-world vaccine effectiveness to accumulate evidence to inform policy and risk communication. This was especially true in Japan and the Philippines, given historical issues that affected vaccine confidence.

**Context:**

Neither country had public health surveillance that could be enhanced to evaluate vaccine effectiveness or readily available national vaccination databases.

**Action:**

Study groups were established in multiple health-care facilities in each country to assess vaccine effectiveness against both symptomatic infection and severe disease.

**Outcome:**

In Japan, multiple study reports were published in Japanese on the web site of the National Institute of Infectious Diseases and presented at the national government’s advisory board. Nationwide media coverage facilitated transparency and increased the confidence of the government and the public in the vaccination programme. In the Philippines, the launch of the study was delayed so as to align the research plan with the interests of various stakeholders and to obtain institutional review board approval. Ultimately, the studies were successfully initiated and completed.

**Discussion:**

There were four main challenges in conducting our studies: finding health-care facilities for data collection; obtaining exposure (vaccination) data; identifying epidemiological biases and confounders; and informing policy and risk communication in a timely manner. Preparedness during inter-emergency/epidemic/pandemic periods to rapidly evaluate relevant interventions such as vaccination is critical and should include the following considerations: (1) the establishment and maintenance of prospective data collection platforms, ideally under public health surveillance (if not, clinical research networks or linked databases); (2) uniform and practical protocols considering biases and confounders; and (3) communication with stakeholders including institutional review boards.

## PROBLEM

COVID-19, caused by severe acute respiratory syndrome coronavirus 2 (SARS-CoV-2), has resulted in substantial morbidity and mortality globally. Once vaccines were rolled out, real-world vaccine effectiveness (VE) data were needed to accumulate evidence to inform policy and risk communication. (*1*) This became more apparent during the early unblinding of randomized controlled trials, (*2*) together with evidence of waning immunity and the emergence of variants with immune escape properties. (*3*, *4*) Although the World Health Organization (WHO) did not recommend that all countries conduct VE studies on account of methodological complexity and susceptibility to biases, (*5*) it was considered valuable for Japan and the Philippines to conduct VE studies for several reasons: (1) historical issues with vaccine confidence in both countries and in neighbouring countries (especially given previous issues that affected vaccine confidence, for example, human papillomavirus and influenza vaccines in Japan (*6*, *7*) and dengue vaccine in the Philippines (*8*)); (2) new vaccine technologies, such as messenger ribonucleic acid (mRNA) vaccines and viral vector vaccines, were rolled out to the general population for the first time and the effects may vary by population subgroup; (3) substantial variation in public health and social measures implemented among countries (which may affect VE estimates (*9*)); and (4) considerable cumulative burden of infections among different populations (as individuals with prior infection are at least partially protected against subsequent infections and diseases). VE studies in low- and middle-income countries (LMICs) were considered particularly informative for the following reasons: (1) evaluation of vaccines that are mainly distributed in LMICs as part of public health response measures; (2) confirmation that the vaccines remain active through distribution networks (for example, no cold chain breach, as temperature control is especially important for vaccines such as mRNA vaccines); and (3) capacity-building to conduct operational research to inform various public health responses for COVID-19 as well as future epidemics and pandemics.

The authors, together with collaborators established health-care facility-based study groups in Japan and the Philippines to assess VE against symptomatic infection (FASCINATE study) and severe disease (MOTIVATE study). (*9*-*15*) This report describes the experience of planning, establishing and executing these VE studies during the COVID-19 pandemic.

## CONTEXT

**Fig. 1:**
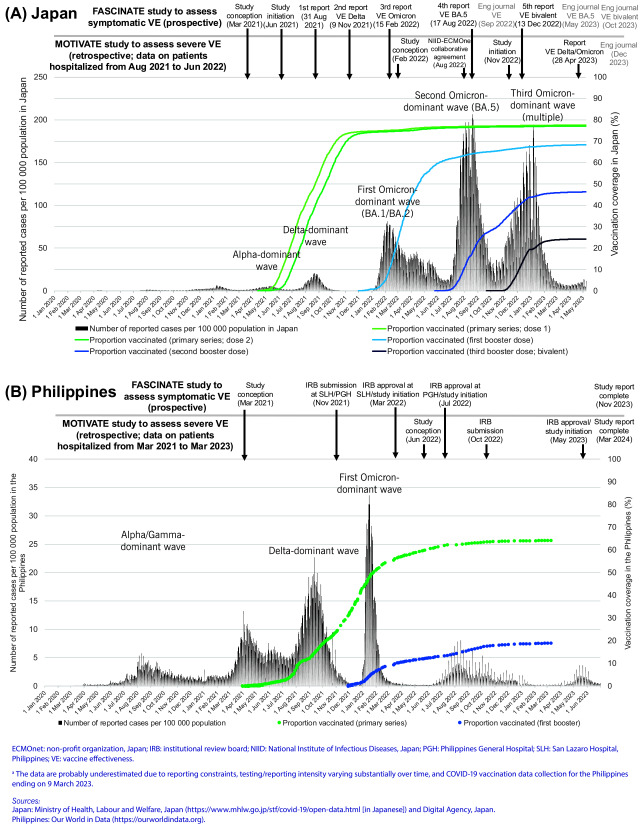
Epidemic curves of the number of reported COVID-19 cases and vaccine rollout with study milestones in (A) Japan and (B) the Philippines^a^

As in other countries, the COVID-19 pandemic substantially affected Japan and the Philippines. The epidemic curve of reported COVID-19 cases and vaccination rollout with selected study milestones in each country are illustrated in **Fig. 1**. In Japan, the primary series rollout started in mid-February 2021, with the first booster dose in December 2021, the second booster dose in May 2022 and the third booster dose (bivalent vaccines) in September 2022. The second booster dose was administered exclusively to individuals who were ≥ 60 years old, had comorbidities or were health-care or long-term care workers. The majority of the administered vaccines were manufactured by Pfizer-BioNTech and Moderna (99.9% for the primary series).

In the Philippines, the primary series rollout started in March 2021. The first booster dose rollout started in November 2021 among health-care workers (HCWs), senior citizens and immunocompromised individuals, and was expanded to adults aged ≥ 18 years in December 2021. The second booster dose rollout started in April 2022 among HCWs and individuals who were ≥ 60 years old, and in July 2022 among individuals who were ≥ 50 years old and those aged 18–49 years with comorbidities. In the FASCINATE study, among the vaccinees for the primary series, 39% received AstraZeneca, 37% received Sinovac, 18% received Pfizer-BioNTech or Moderna, and 6% received other types. Over 90% of the vaccinees received Pfizer-BioNTech or Moderna booster doses.

[**insert** Figure 1]

Existing public health surveillance, such as for influenza-like illness (ILI) and severe acute respiratory infection (SARI), was not easy to enhance rapidly to evaluate VE. Therefore, we collaborated with health-care facilities to set up prospective studies in both countries.

## ACTION

Study groups to assess VE against symptomatic infection (FASCINATE study groups) were formed in each country. Mild symptomatic infection was the outcome of choice, as it was the endpoint of the trials. Health-care facilities that routinely testing for SARS-CoV-2 among symptomatic individuals of different ages in the outpatient setting were recruited and the studies were initiated in each country. The FASCINATE study also aimed to elucidate sociobehavioural factors associated with SARS-CoV-2 infection. Subsequently, emerging evidence suggesting that VE wanes against mild symptomatic infection and is also less effective in the Omicron setting resulted in the need to evaluate VE against severe disease. Therefore, additional MOTIVATE study groups were formed and initiated in both countries. For MOTIVATE study groups, health-care facilities that routinely admitted individuals with COVID-19 and pneumonia due to other etiologies (for example, bacterial pneumonia) were recruited. We examined VE against various severe outcomes, including oxygen use, invasive mechanical ventilation use and death. We also collected data on whether medical intervention, such as oxygen use, was due to COVID-19 or other diseases among those who tested positive for SARS-CoV-2.

## OUTCOME

In Japan, the study prompted the publication of multiple study reports in Japanese on the National Institute of Infectious Diseases (NIID) web site. They were also presented at the national government’s advisory board to inform policy and risk communication (**Fig. 1**). Since NIID is part of the Ministry of Health, Labour and Welfare (MHLW) in Japan, authorization was obtained from the MHLW before publication. Published findings were disseminated via multiple nationwide news media platforms, increasing the confidence of the government and the public in the vaccination programme. This continued until the transition to the endemic phase in May 2023. In the Philippines, due to the delay in initiating the study, the report became available in November 2023 for the FASCINATE (outpatient) study and in March 2024 for the MOTIVATE (inpatient) study.

## Discussion

Many challenges in conducting VE studies were encountered in both countries (**Table 1**). Here, four main challenges are highlighted. The first challenge was identifying health-care facilities willing to participate in the study. HCWs were working around the clock in response to the pandemic, and any additional work was often not possible. In Japan, the authors contacted the health-care facilities directly to seek cooperation. In total, 16 clinics and hospitals for the FASCINATE study and 29 hospitals for the MOTIVATE study agreed to join. Specifically, for the MOTIVATE study in Japan, NIID and ECMOnet (a non-profit organization formed by critical care physicians) successfully collaborated to identify health-care facilities. (*13*) In the Philippines, the FASCINATE study was conducted in two hospitals, while the MOTIVATE study was a single-centre study.

**Table 1:** Implementation challenges in conducting VE studies during the COVID-19 pandemic based on experience in Japan and the Philippines

Implementation challenges	Solutions/mitigations (checkmark [✓] for the ones used and arrowhead [⮚] for suggestions for future studies)	Countries
**Recruitment of health-care facilities**	**Search for health-care facilities where testing is done frequently and where patients with COVID-19 and other respiratory infections are admitted frequently** **Convey the public health value of the research.** **Collaborate with existing clinical networks.** **Design the study in a way that the burden of health-care facilities is minimal.** **Establish a unified database that can link vaccination records and outcomes may ** **minimize/eliminate the need to do this.**	**Both (especially Japan)**
**Unavailability of vaccination record database**	**Refer to either vaccination card or medical chart (if neither is available, self-report).** **Establish a unified database for vaccination records.**	**Both**
Epidemiological biases and confounders (see **Table 2** for a specific list)	**Be careful and agile in consideration of biases and confounders.** **Ensure clear case definition and collection of essential information such as relevant ** **potential confounders (best done as a prospective study by ideally incorporating into public health surveillance, such as ILI or SARI surveillance).** Prepare uniform and practical protocols that can be adopted rapidly if a health emergency occurs. See **Table 2** for specific solutions/mitigations for each bias or confounder.	**Both**
**Timeline**	**Communicate with various stakeholders including institutional review board secretariat/members regularly.** **Establish/maintain platforms such as clinical research networks and unified databases during inter-emergency/epidemic/pandemic period.** **Conduct studies as public health activities rather than research (if feasible under local circumstances).** **Append VE evaluation component to existing public health surveillance such as ILI or SARI surveillance.** **Prepare uniform and practical protocols that can be pre-approved and then rapidly adopted when a health emergency occurs.** **Establish a mechanism to publish and disseminate study results rapidly.**	**Both (especially the Philippines)**
**Maintaining motivation of health-care facilities**	**Periodically communicate and publish findings to acknowledge contributions.**	**Japan**
**Infection prevention and control measures in health-care facilities**	**Show evidence that the virus can be inactivated on paper after several days.** **Design the study in a way that the burden of health-care facilities is minimal.**	**Both**
**Human resources**	**Seek support from medical students who are eager to gain research experience.** **Establish ways to build surge capacity.**	
**Funding**	**Publishing multiple reports resulted in further funding (Japan).** **The World Health Organization provided funding (the Philippines).**	**Both**

ILI: influenza-like illness; SARI: severe acute respiratory infection; VE: vaccine effectiveness.

The second challenge was that there was no national database of vaccination records. Therefore, such data were collected at each health-care facility (using either a vaccination card, medical chart or self-report (*16*)). However, collecting accurate vaccination histories can be resource-intensive, as described in this report. This was a disadvantage compared to some other countries, such as United Kingdom of Great Britain and Northern Ireland, where such data were readily available. However, we saw this as an opportunity to assess VE in an accurate manner by prospectively collecting data that were not readily available and by being able to set a clear clinical case definition to reduce bias caused by unclear definitions. Specifically, for the FASCINATE study, we collected past behavioural data such as attendance at social gatherings that could potentially have been associated with both exposure (for example, the likelihood of vaccination or change in behaviour post-vaccination) and outcome (the likelihood of infection). In fact, the FASCINATE study also aimed to elucidate sociobehavioural factors associated with SARS-CoV-2 infection, which turned out to be important in adjusting for potential biases. (*9*) For the MOTIVATE study, we collected data on whether medical intervention, such as oxygen use, was due to COVID-19 or other diseases among those who tested positive for SARS-CoV-2, (*13*) since incidental infection found at the time of hospital admission with unrelated conditions was an issue in using a database to conduct VE studies. (*17*)

The third challenge was that of evolving epidemiological biases and confounders (**Table 2**). Due to the prospective nature of the study, we were able to mitigate the majority of these, but the risk of residual bias was considered high in the Philippines study results. A reason for this included the likelihood that most unvaccinated individuals were infected before the study’s initiation (which was immediately after the first Omicron surge, which probably afforded better protection compared to vaccination several months earlier, and differential sociodemographic and risk behaviour status between the vaccinated and the unvaccinated.

**Table 2:** Biases and how to approach them in conducting VE studies during the COVID-19 pandemic based on experience in Japan and the Philippines

Epidemiological biases and confounders	Problem	Approach to reduce biases/confounders (checkmark [✓] for the ones used and arrowhead [⮚] for suggestions for future studies)
**Potential confounding factors known at the beginning of the study**	**Potential confounding factors include age, sex, race/ethnicity, socioeconomic status, occupation, chronic medical conditions, close contact history, onset date, and priority groups for vaccination.**	**Adjust for confounders.**
**Diagnostic bias**	**Health workers are more likely to test certain populations such as unvaccinated individuals or individuals at high risk of severe COVID-19.**	**Ask health workers to decide not to test based on vaccination or other status.** **Use specific case definition for study inclusion.**
**Misclassification of the outcome**	**False positives and false negatives**	**Use PCR that has high sensitivity and specificity.** **Use more specific and severe outcomes.** **Restrict to individuals with symptom onset within 2 weeks.**
**Misclassification of the exposure**	**Wrong vaccination data**	**Ascertain vaccination history with vaccine card/certificate.** **Establish a unified database that can link vaccination records and outcomes such as hospitalizations.**
**Prior infection**	**Prior infection may partially protect against subsequent infection, resulting in an underestimate of VE.** **Individuals with known prior SARS-CoV-2 infection are less likely to get vaccinated.**	**Adjust for prior infection.** **Perform sensitivity analysis excluding those with prior SARS-CoV-2 infection.** **Account for underascertainment of prior infection, which can result in residual bias (exploratory use of infection-specific serology may help mitigate this).**
**Spurious waning**	**An ever-increasing pool of unvaccinated individuals become immune through infection, resulting in a progressively increasing underestimate of VE, giving the appearance of waning.**	**Conduct the study in a short period of time before the epidemic peak.** **Enrol only those without prior infection.**
**Vaccination certificate/passport policy (for domestic purposes)**	**Vaccination passports allow vaccinated individuals to engage in high-risk behaviours, such as going to restaurants and bars, while keeping unvaccinated individuals from such activities, resulting in an underestimate of VE (or even negative VE).**	**Adjust for risk behaviour status.**
**Differential risk behaviour based on vaccine status**	**Vaccinated individuals are more likely to engage in high-risk behaviours, such as going to restaurants and bars, as they feel protected, resulting in an underestimate of VE (or even negative VE).**	**Adjust for risk behaviour status.**
**Incidental infection among individuals hospitalized with unrelated conditions**	**If SARS-CoV-2 testing at hospital admission is done for individuals without COVID-19-like symptoms in the setting of high transmission, this will result in an underestimate of VE (given lower VE against infection compared to hospitalization).**	**Use more specific and severe outcomes, such as oxygen or mechanical-ventilation use or, ideally, to restrict individuals who are hospitalized specifically for COVID-19.**
**Care-seeking and testing behaviour; changes in testing strategies**	**Vaccinated persons are less likely to seek care/testing for COVID-19-like illness due to the perception of protection, resulting in an overestimate of VE.** **Changing testing strategies (e.g. after Omicron, testing became less frequent in many countries) can affect results.**	**Breakthrough infection is common enough that individuals should be encouraged to get tested even after vaccination.** **Make sure the testing strategy remains stable (such as by prospective study design).**
**Bias due to co-circulation of influenza, RSV or *Streptococcus pneumoniae* and COVID-19**	**Co-circulation of influenza, RSV or *Streptococcus pneumoniae* and COVID-19 can result in biased VE estimates as propensity to get vaccinated may be similar for COVID-19 vaccines and influenza/pneumococcal vaccines.**	**Exclude influenza/RSV/*Streptococcus pneumoniae* cases or adjust for influenza vaccination/pneumococcal vaccination status.**
**Other residual confounders and biases**	**Other potential residual confounders and biases.**	**Restrict the study population to a particular population group, such as health-care workers, whose sociodemographic factors are similar between the vaccinated and unvaccinated.**

PCR: polymerase chain reaction; RSV: respiratory syncytial virus; VE: vaccine effectiveness.

The final challenge was the timeline. There was a substantial delay in study initiation in the Philippines. What took time was the alignment with various stakeholders and institutional review board (IRB) approval. Following IRB approval, a memorandum of agreement as well as a non-disclosure agreement needed to be signed and validated by the hospital’s legal department with apostille required. Recruitment was also a challenge, as the investigation started right after the Omicron surge. In Japan, we were able to initiate the study and publish reports in a relatively timely manner to inform policy and risk communication. However, it was not always possible to respond to the rapidly evolving policy and communication needs, especially on VE against severe disease.

For future epidemics and pandemics, preparedness during the inter-epidemic/inter-pandemic periods will be critical so that interventions such as vaccination can be rapidly evaluated when such health emergencies occur. Based on our experience, we summarized three main lessons learned. First is the importance of establishing and maintaining platforms to rapidly evaluate interventions such as vaccination. Ideally, these would be incorporated into public health surveillance (for example, ILI or SARI surveillance) and carried out via prospective data collection. The prospective approach would ensure a clear case definition and collection of essential information, such as relevant potential confounders. If this is not feasible, clinical research networks such as the International Severe Acute Respiratory and emerging Infection Consortium and/or a unified database that can link exposure and outcome data (as well as genomic characterization of infections) may be considered. Specifically, setting up these platforms and monitoring epidemics, such as seasonal influenza and respiratory syncytial virus infection, during the inter-emergency/pandemic period in advance is critical, as these can rapidly be applied to newly emerging respiratory infections with pandemic potential. The second lesson is the usefulness of uniform and practical protocols with careful and agile consideration of biases and confounders to conduct clinical research based on policy and risk communication needs, which would also allow for cross-comparison of studies. A guidance document on VE studies was published by WHO, (*5*, *17*) but it was generic in nature. Therefore, some of the authors at the WHO Regional Office for the Western Pacific prepared a practical protocol, which was used as a basis for a VE study in Viet Nam. The third lesson is the value of communication with all potential stakeholders including IRBs during the inter-emergency/pandemic period to pre-approve generic clinical study protocols that can then be expedited when a health emergency occurs, although incorporating VE evaluation into public health surveillance may eliminate this need.

During health emergencies, responding to the event itself is the priority, and conducting operational studies may seem less important. However, evidence-based decision-making is key to a successful response, and such studies are exactly what inform health emergency response.
